# Long‐term nitrogen enrichment mediates the effects of nitrogen supply and co‐inoculation on a viral pathogen

**DOI:** 10.1002/ece3.8450

**Published:** 2022-01-15

**Authors:** Casey A. Easterday, Amy E. Kendig, Christelle Lacroix, Eric W. Seabloom, Elizabeth T. Borer

**Affiliations:** ^1^ Department of Ecology, Evolution, and Behavior University of Minnesota St. Paul Minnesota USA; ^2^ Present address: Carlson School of Management University of Minnesota Minneapolis Minnesota USA; ^3^ Present address: Pathologie Végétale INRAE Montfavet France

**Keywords:** *Avena sativa*, Barley and Cereal Yellow Dwarf Viruses, long‐term N enrichment, plant–pathogen interactions, soil microbes, vectored plant pathogen

## Abstract

Host nutrient supply can mediate host–pathogen and pathogen–pathogen interactions. In terrestrial systems, plant nutrient supply is mediated by soil microbes, suggesting a potential role of soil microbes in plant diseases beyond soil‐borne pathogens and induced plant defenses. Long‐term nitrogen (N) enrichment can shift pathogenic and nonpathogenic soil microbial community composition and function, but it is unclear if these shifts affect plant–pathogen and pathogen–pathogen interactions. In a growth chamber experiment, we tested the effect of long‐term N enrichment on infection by Barley Yellow Dwarf Virus (BYDV‐PAV) and Cereal Yellow Dwarf Virus (CYDV‐RPV), aphid‐vectored RNA viruses, in a grass host. We inoculated sterilized growing medium with soil collected from a long‐term N enrichment experiment (ambient, low, and high N soil treatments) to isolate effects mediated by the soil microbial community. We crossed soil treatments with a N supply treatment (low, high) and virus inoculation treatment (mock‐, singly‐, and co‐inoculated) to evaluate the effects of long‐term N enrichment on plant–pathogen and pathogen–pathogen interactions, as mediated by N availability. We measured the proportion of plants infected (i.e., incidence), plant biomass, and leaf chlorophyll content. BYDV‐PAV incidence (0.96) declined with low N soil (to 0.46), high N supply (to 0.61), and co‐inoculation (to 0.32). Low N soil mediated the effect of N supply on BYDV‐PAV: instead of N supply reducing BYDV‐PAV incidence, the incidence increased. Additionally, ambient and low N soil ameliorated the negative effect of co‐inoculation on BYDV‐PAV incidence. BYDV‐PAV infection only reduced chlorophyll when plants were grown with low N supply and ambient N soil. There were no significant effects of long‐term N soil on CYDV‐RPV incidence. Soil inoculant with different levels of long‐term N enrichment had different effects on host–pathogen and pathogen–pathogen interactions, suggesting that shifts in soil microbial communities with long‐term N enrichment may mediate disease dynamics.

## Graphical abstract

Soil microbes alter plant nutrient supply and plant defenses, both of which can modify plant‐pathogen and pathogen‐pathogen interactions. It is unclear if changes in soil microbial communities exposed to long‐term nitrogen (N) enrichment affect their mediation of plant diseases. In a laboratory experiment, soil exposed to long‐term N enrichment neutralized the negative effects of nitrogen and co‐inoculation on infection incidence of a grass virus.

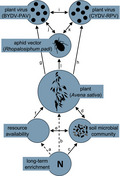

## 1 INTRODUCTION

Fossil fuel use and fertilizer production have more than doubled reactive nitrogen (N) inputs to terrestrial ecosystems since pre‐industrialization (Galloway et al., 2004; Vitousek et al., 1997). Nitrogen enrichment can profoundly impact terrestrial plant systems, increasing productivity and reducing biodiversity (Elser et al., 2007; Midolo et al., 2019). Furthermore, the effects of N enrichment on plants can have repercussions throughout food webs (He & Silliman, 2015; Ritchie, 2000; Sedlacek et al., 1988). For example, N enrichment can modify plant–pathogen interactions (Dordas, 2009; Veresoglou et al., 2013) and interactions among different pathogens that co‐infect plants (Kendig et al., 2020; Lacroix et al., 2014; Strauss et al., 2021). The communities of pathogens that rely on plants can in turn impact plant productivity, community composition, and ecosystem processes (Borer et al., 2021; Lovett et al., 2010; Paseka et al., 2020). However, a key component of terrestrial systems—soil microbes (e.g., bacteria, fungi, archaea)—have been neglected in many studies of N enrichment on aboveground plant pathogens. Soil microbes affect plant access to N (Kuzyakov & Xu, 2013; van der Heijden et al., 2008) and plant interactions with above‐ and belowground pathogens (Berendsen et al., 2012; van Loon et al., 1998). Therefore, laboratory‐based studies that do not include natural soil microbial communities may under‐ or overestimate the effects of N enrichment on plant–pathogen interactions under field conditions. Moreover, N enrichment can change the nature of interactions between soil microbes and plants through time (Huang et al., 2019; Johnson, 1993; Keeler et al., 2008; Weese et al., 2015). Consequently, even studies that include natural soil microbial communities may mischaracterize the impacts of N enrichment on plant pathogen communities if they do not encompass long enough time scales.

**FIGURE 1:**
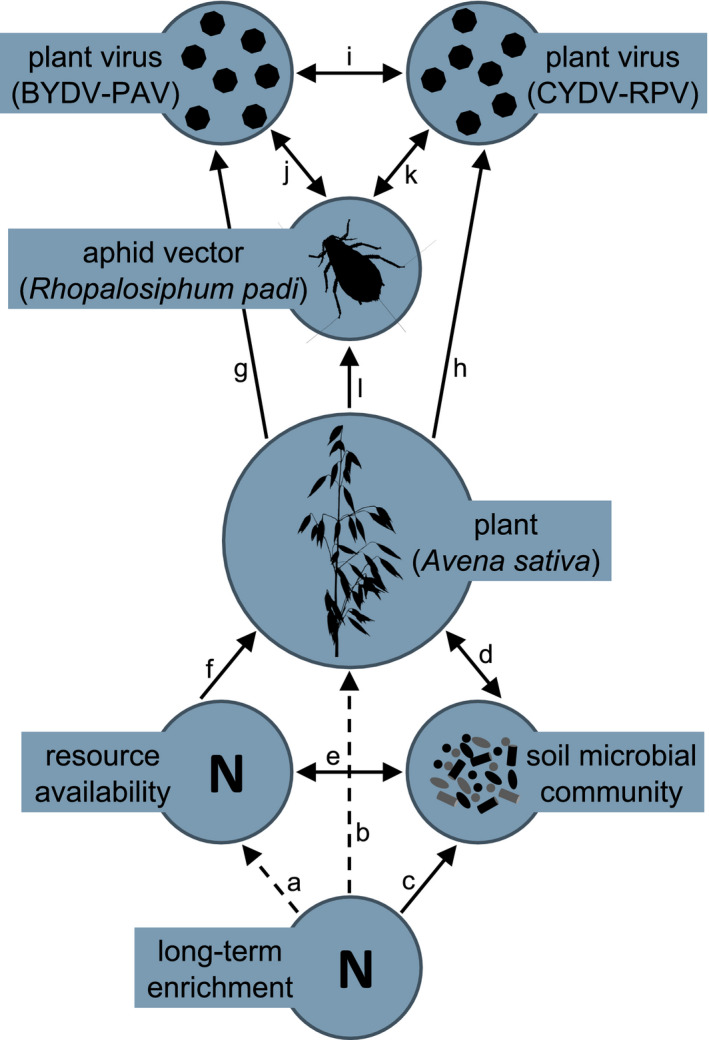
Hypothesized interactions in terrestrial plant–pathogen systems. Long‐term N enrichment increases N availability (a), shifts plant community productivity and composition over time (b), and alters the structure and function of soil microbial communities (c). We isolated the effects of long‐term N fertilization on plant diseases through changes in the soil microbial community by inoculating growing medium with soil obtained from a long‐term N fertilization experiment, therefore, removing the effects of long‐term N fertilization on contemporary N availability (a) and the plant community (b; indicated by dashed lines). Soil microbes can affect plant diseases by inducing plant defenses (d) and suppressing soil‐borne pathogens (not shown). We hypothesize that soil microbes also affect plant diseases by altering contemporary N availability (e), which affects plant diseases by increasing plant growth or defenses or mediating pathogen replication (f). Although each connection (a–f) has been demonstrated, it is unclear if the effects of long‐term N enrichment on soil microbial can mediate plant–pathogen (g, h) or pathogen–pathogen (i) interactions. In plant–virus systems, insect vectors are important determinants of infection and co‐infection (j, k) and can be affected by soil properties (i.e., N availability, microbial communities), mediated by the plant (l). *Avena sativa* icon by Andreas Trepte, vectorized by T. Michael Keesey (https://creativecommons.org/licenses/by‐sa/3.0/)

While it is widely demonstrated that N can impact plant–pathogen interactions (Dordas, 2009; Lekberg et al., 2021; Veresoglou et al., 2013), the specific pathway is not typically understood, and soil microbes may play an important role (Figure 1). Nitrogen is an essential component of genetic material and proteins (Sterner & Elser, 2002). Changes in N availability can, therefore, modify the fitness of plants (Johnson, 1993; Welch & Leggett, 1997), microbes (Kuzyakov & Xu, 2013; Schimel & Bennett, 2004), and insect vectors of plant pathogens (Bogaert et al., 2017; Nowak & Komor, 2010). Because plants, their pathogens, and insect vectors rely on N, N enrichment can increase or decrease infection prevalence (Borer et al., 2014; Seabloom et al., 2010), pathogen load (Fagard et al., 2014; Hoffland et al., 2000; Mitchell et al., 2003; Robert et al., 2004; Singh, 1970; Whitaker et al., 2015), and disease resistance (Bellin et al., 2013; Dietrich et al., 2004; Mur et al., 2016). Furthermore, individual plants and plant communities frequently host multipathogen communities (Bass et al., 2019; Seabloom et al., 2009) which may shift in composition with N enrichment (Kendig et al., 2020; Lacroix et al., 2014; Strauss et al., 2021). Soil microbes and N enrichment may interact to affect plant–pathogen and pathogen–pathogen interactions. For example, fertilizer and green manure can increase the disease‐suppressive activity of foliar and rhizosphere microbial communities (Berg & Koskella, 2018; Wiggins & Kinkel, 2005).

Soil microbes may mediate plant–pathogen interactions through multiple mechanisms, including altering the amount of N available to the plant. Soil microbes can increase plant access to N through N fixation, N mineralization, and extending root networks (van der Heijden et al., 2008), and they also may compete with plants for N (Kuzyakov & Xu, 2013; Schimel & Bennett, 2004). N enrichment can reduce the benefits plants receive from microbial mutualists (Johnson, 1993; Weese et al., 2015), although losses can be offset by the direct benefits of bioavailable N (Farrer & Suding, 2016; Johnson, 1993). N enrichment has variable effects on N mineralization rates (Chen et al., 2019; Mueller et al., 2013), mediated by changes in soil microbial community composition and pH (Chen et al., 2019). Over decadal time scales, N enrichment can drive compositional and evolutionary changes in soil microbial communities that affect their N‐related interactions with plants (Huang et al., 2019; Klinger et al., 2016). For example, N enrichment can shift the relative abundance of archaea and bacteria that oxidize ammonia, depending on the form of N added (Leff et al., 2015; Moreau et al., 2015).

Soil microbial communities also may mediate plant–pathogen interactions by varying in abundance of soil‐borne plant pathogens or inducing host defenses (Mauch‐Mani et al., 2017; Schlatter et al., 2017). For example, soil microbes can suppress soil‐borne pathogens via competition for resources, interference with pathogen signaling, or production of antibiotic compounds and lytic enzymes (Berendsen et al., 2012; Lugtenberg & Kamilova, 2009). In addition, beneficial and pathogenic soil microbes can induce disease resistance pathways in plants, priming them for faster and stronger responses to aboveground pathogen attacks (Mauch‐Mani et al., 2017; Pieterse et al., 2014; van Loon et al., 1998). Soil biota associated with induced disease resistance include bacteria in the genera *Pseudomonas*, *Serratia*, and *Bacillus* and fungi in the genera *Trichoderma*, *Fusarium*, *Piriformospora*, and *Glomeromycota* (Mauch‐Mani et al., 2017; Pieterse et al., 2014). Nitrogen enrichment may modify the effects of soil microbes on plant diseases through compositional or evolutionary shifts in microbial communities or changes in microbe–pathogen interactions (Huang et al., 2019; Klinger et al., 2016; Otto‐Hanson et al., 2013; Schlatter et al., 2013). For example, N enrichment can reduce the abundance and colonization rates of arbuscular mycorrhizal fungi (AMF), which includes the genus *Glomeromycota* (Jia et al., 2020; Leff et al., 2015; Treseder, 2004). In contrast, N enrichment tends to increase phyla and classes containing some groups of fungi (*Trichoderma* and *Fusarium*) and bacteria (*Pseudomonas*, *Serratia*, and *Bacillus*) that induce disease resistance (Chen et al., 2019; Fierer et al., 2012; Leff et al., 2015; Ramirez et al., 2012).

Long‐term N enrichment may impact plant–pathogen and pathogen–pathogen interactions indirectly through changes in the soil microbial community via three main processes: altered N availability, induced disease resistance, and changes in abundance of soil pathogens (Figure 1). Although soil pathogen impacts on plants have received substantial attention (Kuzyakov & Xu, 2013; Mauch‐Mani et al., 2017; van der Heijden et al., 2008), the effects of long‐term N enrichment, as mediated by nonpathogenic soil microbes, on aboveground plant pathogens are not well understood. To fill this gap, we evaluated the effects of soil microbial communities from a long‐term N enrichment study on plant–pathogen and pathogen–pathogen interactions using two widespread and economically important insect‐vectored plant viruses (BYDV‐PAV and CYDV‐RPV). We used aphids (*Rhopalosiphum padi*) to inoculate oat plants (*Avena sativa*) with these Barley and Cereal Yellow Dwarf Viruses (B/CYDVs) across a full factorial combination of soil inoculum from a long‐term N enrichment experiment (ambient, low, or high N soil and a noninoculated control), N supply rates (low or high), and virus inoculation treatments (mock‐, single‐, co‐inoculation). For each treatment, we measured virus incidence (i.e., the proportion of plants that became infected), plant biomass, and leaf chlorophyll content. We addressed three questions: (1) What are the effects of N on single infection and co‐infection incidence in plants grown in noninoculated growing medium? (2) Do long‐term N‐enriched soils mediate the effects of N on single infection and co‐infection incidence? (3) Do long‐term N‐enriched soils mediate the effects of N or infection on host traits? We hypothesized that inoculating growing medium with soils exposed to different levels of N enrichment, which likely differ in soil microbial community structure or function, would differentially affect host–pathogen and pathogen–pathogen interactions.

## 2 MATERIALS AND METHODS

### 2.1 Study system

The B/CYDVs cause systemic infections in over 150 grass species in the Poaceae family, stunting growth, yellowing or reddening leaves, and reducing fecundity (Carrigan et al., 1983; D’Arcy & Burnett, 1995; Irwin & Thresh, 1990). B/CYDVs comprise members of the *Luteovirus* (BYDVs) and *Polerovirus* (CYDVs) genera (Miller et al., 2002). BYDV‐PAV and CYDV‐RPV are considered representative members of each genera and have been the foci of many studies (Kendig et al., 2020; Lacroix et al., 2014; Power et al., 1991; Seabloom et al., 2009). These viruses are transmitted by a range of aphid vectors, including *R*. *padi* that transmits both BYDV‐PAV and CYDV‐RPV (D’Arcy & Burnett, 1995). Importantly, these viruses are strictly insect vectored; they cannot be transmitted to plants via soil. We used *A*. *sativa* L. cv. Coast Black Oat as our plant host species. *Avena sativa* can host AMF (Yang et al., 2010) and AMF were found to have a positive effect on BYDV‐PAV titer in *Avena fatua* under elevated CO_2_ (Rúa et al., 2013). Soil was collected from Cedar Creek Ecosystem Science Reserve and Long‐Term Ecological Research site (CDR; see next section). Prior to conducting this work, we established that both of our focal viruses are present in natural communities at CDR. In 2009, we haphazardly collected 153 plants of 10 different grass species across CDR to determine which B/CYDVs were present. We assessed infection status via ELISA as detailed in Seabloom et al. (2009). Overall infection prevalence of BYDV‐PAV was 0.17 and CYDV‐RPV was 0.03.

### 2.2 Long‐term N‐enriched soils

In June 2014, we collected soil cores from a long‐term experiment in a successional grassland at CDR (experiment “E001,” www.cedarcreek.umn.edu; Bethel, MN, USA). Cedar Creek has sandy, N‐limited soils and a background wet N deposition rate of approximately 6 kg N ha^−1^ year^−1^ (58% NH_4_, 42% NO_3_) (Clark & Tilman, 2008; Tilman, 1987). We collected soils from field A, which was abandoned from agriculture in 1968 and burned annually beginning in 2005. These plots had received annual additions of P, K, Ca, Mg, S, and citrate‐chelated trace metals since 1982 and three levels of N fertilizer: 0, 34, or 272 kg N ha^−1^ year^−1^ (see Tilman, 1987 for details). In this experiment, long‐term N enrichment has increased plant biomass and soil N concentration and decreased plant species richness (Isbell, Reich, et al., 2013; Isbell, Tilman, et al., 2013). In addition, N enrichment in this experiment is associated with a shift in bacterial community composition (Fierer et al., 2012) and phenotypes (Schlatter et al., 2013) toward faster growing bacteria with narrower resource niches. We randomly selected three plots for each N enrichment rate (plots 22A, 45A, 54A, 8D, 23D, 38D, 40H, 17H, and 52H) and six locations within each 4 × 4 m plot to extract a soil core (1.9 cm diameter and 10 cm deep). In the lab, soil cores were passed through a 4‐mm sieve, then twice through a 2‐mm sieve to remove coarse debris and roots, and then combined based on their N enrichment rate.

Next, we prepared soil microcosms by filling four large, surface sterilized bins with 17 L of potting soil composed of 70% Sunshine medium vermiculite (vermiculite and <1% crystalline silica; Sun Gro) and 30% Turface MVP (calcined clay containing up to 30% crystalline silica; Turface Athletics), saturated with tap water (approximately 5 L for every 20 L of dry soil) and autoclaved at 121°C and 15 psi for 60 min to kill the naturally existing microbial consortium. We then mixed 350 ml of field soil from each N enrichment level separately into the bins. Field soil comprised approximately 2% of the bin soil volume. We did not mix field soil into the fourth bin. Lastly, we covered the bins with nonairtight lids and incubated the soil at 25°C for 11 days.

### 2.3 Experimental setup and implementation

For each of the four soil microcosms, we filled 80 conical plastic pots (3.8 cm diameter × 21 cm depth, 164 ml) with soil mixture and planted one *A*. *sativa* seed per pot 4.5 cm from the surface of the soil. Seeds were obtained from the USDA (National plant germplasm system, USDA; USA) in June 2013 and were surface sterilized with 12.5% bleach solution. Then, we haphazardly assigned plants to later receive 1 of 2 N supply rates (7.5 μM NH_4_NO_3_ was “low N” and 375 μM NH_4_NO_3_ was “high N”; Table A1) and 1 of 4 virus inoculations (BYDV‐PAV, CYDV‐RPV, co‐inoculation, or mock inoculation), leading to 10 replicates per treatment. Plants grew in a growth chamber containing only healthy plants with a 16:8 h light:dark cycle at 19–20°C under Lumilux high pressure sodium ET18 bulbs for 11 days. Two days after planting, we watered the pots with 30 ml of the modified Hoagland solution (Hoagland & Arnon, 1938; Lacroix et al., 2014; Table A1) corresponding to the plant’s assigned N supply rate. We watered plants with these solutions twice per week until harvest.

When the plants had been growing for 22 days, we used *R*. *padi* aphids to inoculate them with BYDV‐PAV, CYDV‐RPV, both viruses, or to perform mock inoculations. *Rhopalosiphum padi* were obtained from Dr. G. Heimpel at the University of Minnesota (St. Paul, MN, USA) and reared on *A*. *sativa* in growth chamber conditions described above (except with 28W Ultramax EcoXL lights). BYDV‐PAV and CYDV‐RPV isolates were obtained from Dr. S. Gray at Cornell University (Ithaca, NY, USA) in January 2013. They were also maintained in *A*. *sativa* plants in similar growth chamber conditions (except with 40 W cool white light bulbs). We inoculated plants by allowing aphids to feed on either BYDV‐PAV‐ or CYDV‐RPV‐infected *A*. *sativa* tissue in 25‐ml glass tubes sealed with corks for approximately 48 h. Then, we transferred the aphids to 2.5 × 8.5 cm, 118‐μm polyester mesh cages secured to one leaf on each experimental plant with Parafilm and bobby pins. Ten aphids were used to inoculate each plant, with 5 carrying each virus for the co‐inoculation treatment, 5 viruliferous (carrying virus), and 5 nonviruliferous aphids for each single virus treatment, and 10 nonviruliferous aphids for the mock inoculation treatment. We allowed aphids to feed on the experimental plants for approximately 96 h, after which we manually killed all aphids and removed the cages. Plants grew for 19 more days before we took measurements. To estimate N stress through leaf chlorophyll content (Zhao et al., 2015), we took three measurements per plant with a SPAD‐502 Meter (Soil Plant Analysis Development; Konica Minolta). Then, we harvested and weighed the aboveground biomass, which we stored at −20°C until it was analyzed for virus infection.

### 2.4 Detection of B/CYDV infection

To extract total RNA, we ground approximately 50 mg of leaf tissue per plant in a bead‐beater with a copper BB and 1 ml of TRIzol™ Reagent (Invitrogen™, Thermo Fisher Scientific) as per the manufacturer’s instructions. We then purified RNA from the cellular components following the extraction protocol published by Lacroix et al. (2014). We resuspended the purified RNA in nuclease‐free water and stored the samples at −20°C until performing reverse transcription polymerase chain reaction (RT‐PCR). We used a nanodrop spectrophotometer (Thermo Fisher Scientific) to quantitate the concentration of RNA within each sample and then performed a multiplex RT‐PCR assay to isolate and amplify BYDV‐PAV and CYDV‐RPV nucleic acids as published previously (Deb & Anderson, 2008; Lacroix et al., 2014).

**FIGURE A1:**
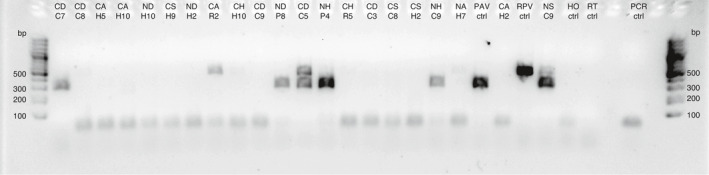
An example gel electrophoresis used to evaluate infections of BYDV‐PAV and CYDV‐RPV following RT‐PCR. A 100 bp DNA ladder is on either side of the samples and controls. BYDV‐PAV is detected at 298 bp and CYDV‐RPV is detected at 447 bp. Labels for experimental samples consist of three letters and a number and follow the format: N supply rate (C = low N, N = high N), field soil inoculation (S = noninoculated growing medium, A = 0 kg N ha^−1^ year^−1^, D = 34 kg N ha^−1^ year^−1^, H = 272 kg N ha^−1^ yr^−1^), virus inoculation (C = co‐inoculation, H = mock‐inoculation, P = BYDV‐PAV, R = CYDV‐RPV), and replicate number. There are positive controls for BYDV‐PAV (PAV ctrl) and CYDV‐RPV (RPV ctrl). There are negative controls for noninoculated *A*. *sativa* (HO ctrl = healthy oat control), reverse transcription components (RT ctrl) and PCR components (PCR ctrl)

We combined 5 μl of each PCR product with 2 μl of 6× loading dye (Genesee Scientific) and loaded the samples and 100 bp DNA ladder (Apex Bioresearch Products) into an Agarose‐1000 gel (Invitrogen, Thermo Fisher Scientific) stained with 2% SybrSafe (Invitrogen, Thermo Fisher Scientific). After 25 min at 120 V, we observed the gel with a UV‐light EZ doc system (Bio‐Rad Laboratories) to detect bands at 298 bp and 447 bp, indicating the presence of BYDV‐PAV and CYDV‐RPV, respectively (Figure A1).

### 2.5 Statistical analyses

We assessed the effects of the experimental treatments on the infection incidence of BYDV‐PAV and CYDV‐RPV using binomial (logit‐link) generalized linear regressions with virus infection as a binary response variable and long‐term N‐enriched soil treatment (noninoculated, ambient N, low N, or high N), N supply (binary variable), whether the plants were co‐inoculated (binary variable), and their interactions as independent variables. The intercepts represented singly inoculated plants grown in noninoculated growing medium with low N supply. We tested the effects of N supply and soil treatment on co‐infection incidence using an analogous procedure. Samples with an infection inconsistent with the inoculation treatment were removed from analyses. Inconsistent infections likely arose from small aphids escaping cages during the inoculation period and occurred in 31 of 229 plants (Table A2). Treatment sample sizes in the final dataset ranged from 7 to 10.

To assess the effects of the experimental treatments on the *A*. *sativa* plants, we used linear regressions with log‐transformed biomass and log‐transformed chlorophyll content as response variables and long‐term N‐enriched soil treatment, N supply, successful inoculation treatment (mock, BYDV‐PAV only, and CYDV‐RPV only), and their interactions as the independent variables. We omitted plants from analyses that were unsuccessfully inoculated, either because the intended infection was not detected or because an unintended infection was detected (Table A2). Co‐infected plants were omitted from analyses due to limited sample sizes. The chlorophyll values used in the model were the averages of three measurements per plant. The intercepts represented mock‐inoculated plants grown in noninoculated growing medium with low N supply. Treatment sample sizes in the final dataset ranged from three to nine.

Regressions described above were fit using Bayesian models with the brms package in R version 4.0.2 (Bürkner, 2017; R Core Team, 2020). Models had three chains of 6000 iterations each with a 1000 iteration discarded burn‐in period. Gaussian distributions with a mean of 0 and a standard deviation of 10 were used as prior distributions for intercepts and coefficients (very weakly informative; McElreath, 2015). We used a half Student’s *t* distribution with 3 degrees of freedom, a location of 0, and a scale of 10 as the prior distribution for the residual standard deviations (Bürkner, 2017). We assessed model fit by ensuring that r‐hat values were equal to one, that the three chains were well mixed, and that simulated data from the posterior predictive distributions were consistent with observed data. In the results, we report point estimates with 95% highest posterior density intervals based on posterior samples of model coefficients in brackets.

To evaluate the effect of sample size on the probability of detecting an effect with quantile‐based 95% credible intervals that omit zero, we simulated 1000 datasets of the same sample sizes and with the mean effect size measured in the experiment. We drew simulated values from normal distributions with means equal to model estimates for each treatment and standard deviations equal to the overall model‐estimated standard deviation. We fit regressions to the simulated datasets and calculated the number of times the 95% credible intervals of the variable of interest omitted zero (Kurz, 2019). We repeated the analysis with multiple sample sizes. We performed this analysis for the effects of CYDV‐RPV infection on log‐transformed plant biomass, where the mean difference was −0.23, the sample sizes were 8 (mock‐inoculated, low N supply, noninoculated growing medium) and 6 (CYDV‐RPV infected, low N supply, noninoculated growing medium), and the regression was a normal linear regression with infection status as the independent variable.

## 3 RESULTS

### 3.1 The effects of N supply on infection incidence in noninoculated growing medium

**FIGURE 2:**
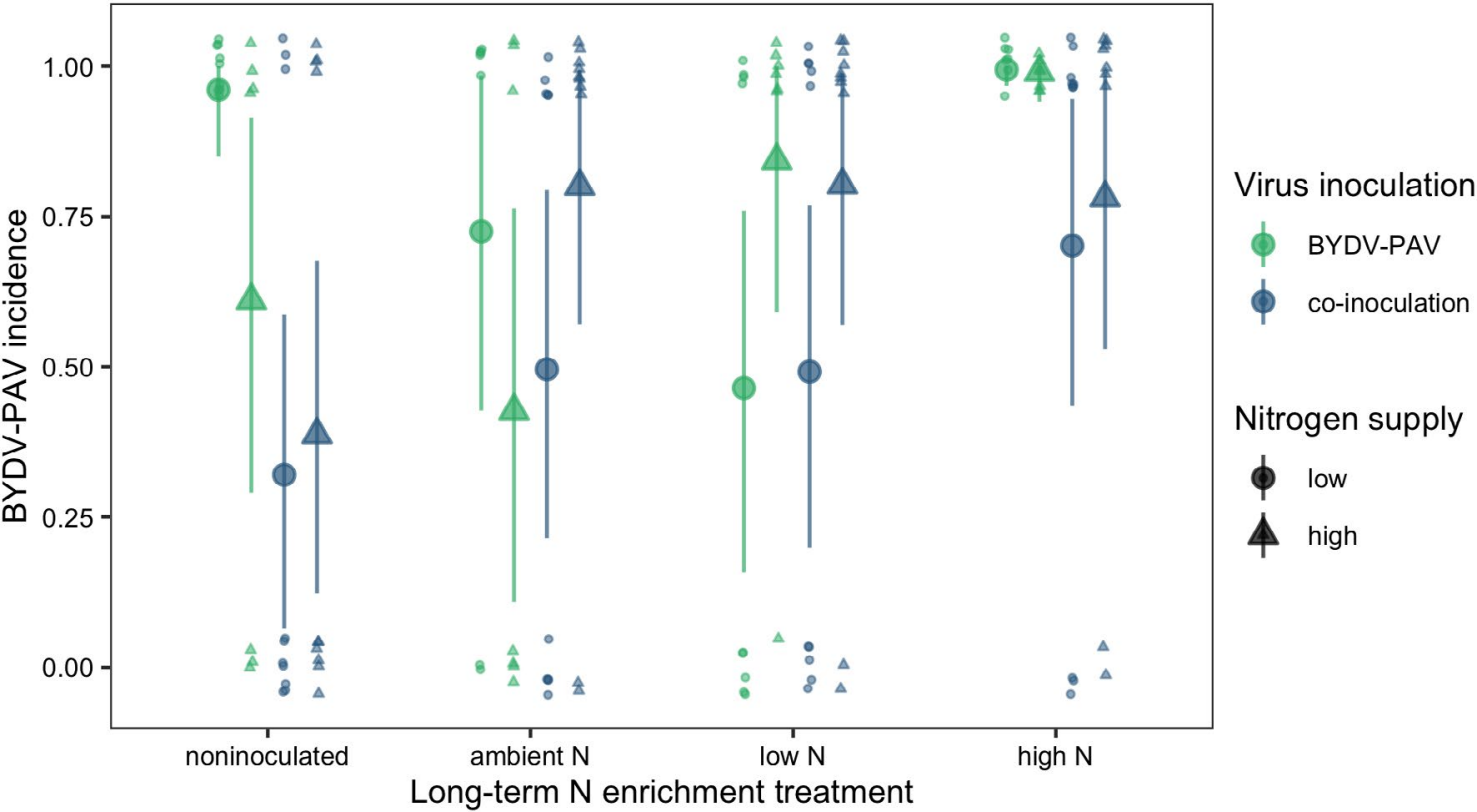
BYDV‐PAV incidence of plants grown with noninoculated growing medium, field soil exposed to long‐term ambient N, field soil exposed to long‐term low N, and field soil exposed to long‐term high N. Plants were grown with low or high N supply and were inoculated either with BYDV‐PAV only or BYDV‐PAV and CYDV‐RPV (co‐inoculated). Points and error bars are model‐estimated mean and 95% highest posterior density intervals (HDI). Small points are raw data, which take on values of either zero or one and are jittered for visualization

**FIGURE 3:**
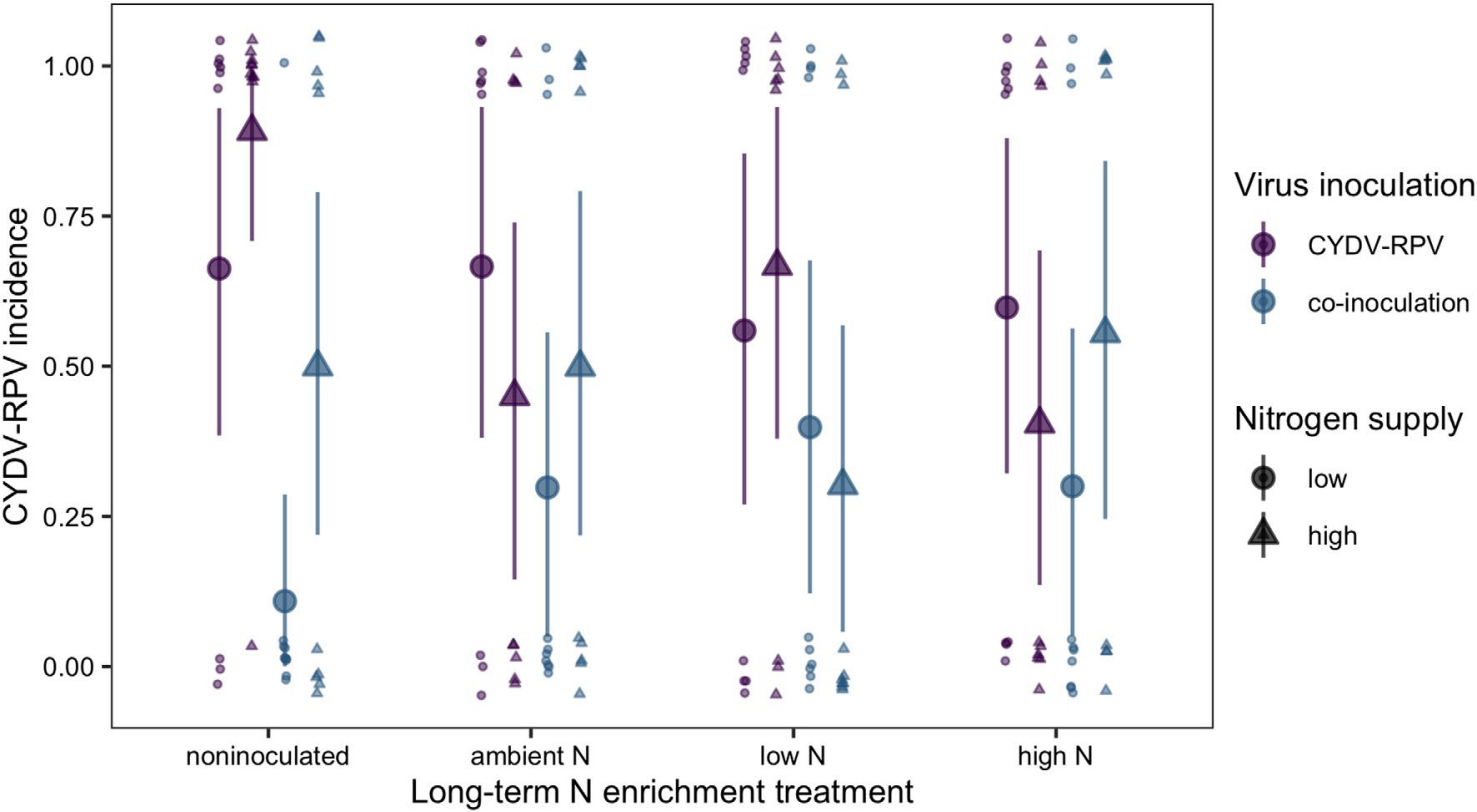
CYDV‐RPV incidence of plants grown with noninoculated growing medium, field soil exposed to long‐term ambient N, field soil exposed to long‐term low N, and field soil exposed to long‐term high N. Plants were grown with low or high N supply and were inoculated either with CYDV‐RPV only or BYDV‐PAV and CYDV‐RPV (co‐inoculated). Points and error bars are model‐estimated mean and 95% HDI. Small points are raw data, which take on values of either zero or one and are jittered for visualization

**FIGURE 4:**
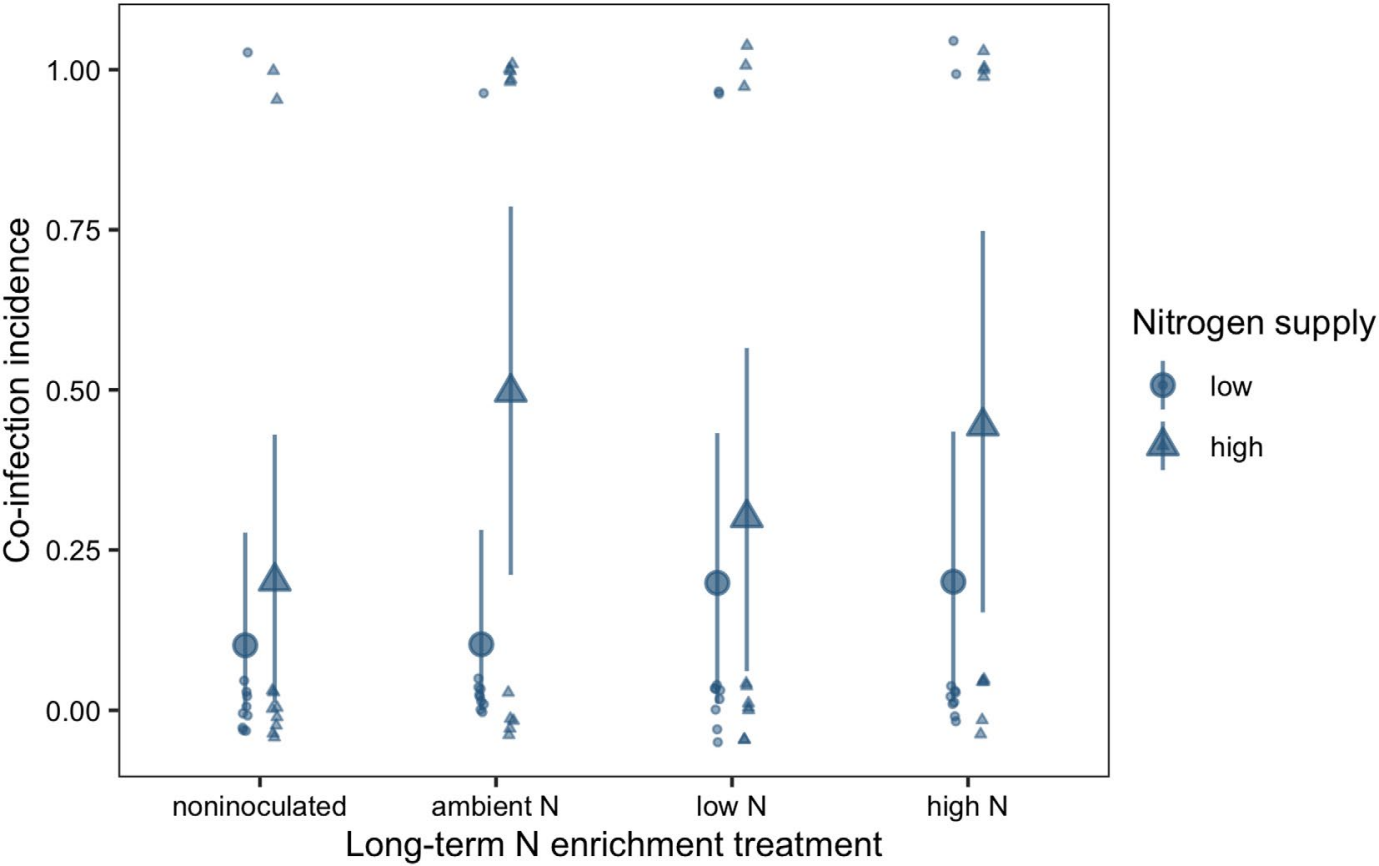
Co‐infection incidence of plants grown with noninoculated growing medium, field soil exposed to long‐term ambient N, field soil exposed to long‐term low N, and field soil exposed to long‐term high N. Plants were grown with low or high N supply and were co‐inoculated with BYDV‐PAV and CYDV‐RPV. Points and error bars are model‐estimated mean and 95% HDI. Small points are raw data, which take on values of either zero or one and are jittered for visualization

The BYDV‐PAV incidence of singly inoculated plants grown with low N supply was 0.96 [0.85, 1.00] (Figure 2). High N supply reduced BYDV‐PAV incidence in singly inoculated plants to 0.61 (−36% [−71%, −4.3%]) and co‐inoculation reduced BYDV‐PAV incidence to 0.32 (−67% [−93%, −39%], Figure 2). However, high N supply did not reduce BYDV‐PAV incidence of co‐inoculated plants (estimated change relative to low N supply: 55% [−83%, 271%]), leading to an interaction between N supply and co‐inoculation (Table 1). CYDV‐RPV incidence of singly inoculated plants grown with low N supply was 0.66 [0.38, 0.93] (Figure 3). Co‐inoculation reduced CYDV‐RPV incidence to 0.11 (−83% [−100%, −53%], Figure 3). Nitrogen supply did not affect CYDV‐RPV incidence in singly or co‐inoculated plants (Table 2). The average co‐infection incidence of co‐inoculated plants grown with low N supply was 0.10 [0.00, 0.28]. Nitrogen supply did not significantly affect co‐infection incidence (Figure 4, Table 3).

**TABLE 1:** Summary of generalized linear regression of BYDV‐PAV incidence (*n* = 139)

Variable^a^	Estimate	Std. error	95% CI		R‐hat
			Lower	Upper	
**Intercept** ^b^	**4.22**	**1.79**	**1.42**	**8.30**	**1.00**
**N supply**	**−3.70**	**1.90**	**−7.94**	**−0.46**	**1.00**
**Co‐inoculation**	**−5.06**	**1.88**	**−9.25**	**−1.92**	**1.00**
Soil ambient N	−3.07	1.96	−7.33	0.37	1.00
**Soil low N**	**−4.38**	**1.89**	**−8.57**	**−1.29**	**1.00**
Soil high N	6.46	4.92	−1.70	17.12	1.00
**N supply × co‐inoculation**	**4.03**	**2.07**	**0.27**	**8.41**	**1.00**
Soil ambient N × N supply	2.21	2.20	−1.83	6.77	1.00
**Soil low N × N supply**	**5.94**	**2.29**	**1.89**	**10.80**	**1.00**
Soil high N × N supply	3.68	5.66	−6.83	15.34	1.00
**Soil ambient N × co‐inoculation**	**3.89**	**2.12**	**0.03**	**8.33**	**1.00**
**Soil low N × co‐inoculation**	**5.18**	**2.07**	**1.58**	**9.72**	**1.00**
Soil high N × co‐inoculation	−4.67	4.96	−15.31	3.63	1.00
Soil ambient N × N supply × co‐inoculation	−0.92	2.55	−6.10	4.04	1.00
Soil low N × N supply × co‐inoculation	−4.62	2.62	−10.03	0.34	1.00
Soil high N × N supply × co‐inoculation	−3.49	5.71	−15.27	7.31	1.00

^a^ Variables with quantile‐based 95% credible intervals that omit zero are in bold.

^b^ Intercept factor levels: low N supply, BYDV‐PAV inoculation, noninoculated growing medium.

**TABLE 2:** Summary of generalized linear regression of CYDV‐RPV incidence (*n* = 154)

Variable^a^	Estimate	Std. error	95% CI		
			Lower	Upper	R‐hat
Intercept^b^	0.76	0.74	−0.62	2.28	1.00
N supply	1.77	1.31	−0.60	4.62	1.00
**Co‐inoculation**	**−3.27**	**1.31**	**−6.12**	**−0.94**	**1.00**
Soil ambient N	0.02	1.04	−2.01	2.08	1.00
Soil low N	−0.49	1.00	−2.48	1.45	1.00
Soil high N	−0.32	1.00	−2.30	1.61	1.00
N supply × co‐inoculation	0.74	1.75	−2.78	4.22	1.00
Soil ambient N × N supply	−2.77	1.64	−6.17	0.31	1.00
Soil low N × N supply	−1.25	1.64	−4.64	1.84	1.00
Soil high N × N supply	−2.64	1.62	−6.02	0.40	1.00
Soil ambient N × co‐inoculation	1.53	1.64	−1.57	4.89	1.00
Soil low N × co‐inoculation	2.55	1.62	−0.46	5.85	1.00
Soil high N × co‐inoculation	1.88	1.63	−1.15	5.19	1.00
Soil ambient N × N supply × co‐inoculation	1.21	2.19	−3.10	5.59	1.00
Soil low N × N supply × co‐inoculation	−1.74	2.22	−6.08	2.63	1.00
Soil high N × N supply × co‐inoculation	1.32	2.21	−3.04	5.72	1.00

^a^ Variables with quantile‐based 95% credible intervals that omit zero are in bold.

^b^ Intercept factor levels: low N supply, CYDV‐RPV inoculation, noninoculated growing medium.

**TABLE 3:** Summary of generalized linear regression of co‐infection incidence (*n* = 79)

Variable^a^	Estimate	Std. error	95% CI		R‐hat
			Lower	Upper	
**Intercept** ^b^	**−2.63**	**1.18**	**−5.34**	**−0.73**	**1.00**
N supply	1.05	1.43	−1.57	4.07	1.00
Soil ambient N	−0.02	1.70	−3.56	3.25	1.00
Soil low N	1.03	1.46	−1.67	4.10	1.00
Soil high N	1.04	1.44	−1.62	4.04	1.00
Soil ambient N × N supply	1.59	1.99	−2.21	5.64	1.00
Soil low N × N supply	−0.39	1.80	−4.06	3.03	1.00
Soil high N × N supply	0.28	1.79	−3.28	3.71	1.00

^a^ Variables with quantile‐based 95% credible intervals that omit zero are in bold.

^b^ Intercept factor levels: low N supply, noninoculated growing medium.

### 3.2 Long‐term N‐enriched soils and N supply: effects on infection incidence

With low N supply, inoculation with low N soil reduced BYDV‐PAV incidence to 0.46 [0.16, 0.76], a 52% decrease [−83%, −20%] relative to plants grown in noninoculated growing medium (Figure 2). In contrast to the negative effect of high N supply when plants were grown in noninoculated growing medium, high N supply did not reduce BYDV‐PAV incidence when plants were grown with low N soil inoculation (estimated change relative to low N supply: 112% [−33%, 316%], Figure 2), leading to an interaction between N supply and low N soil (Table 1). Similarly, high N supply did not affect BYDV‐PAV incidence when plants were grown with ambient N (estimated change relative to low N supply: −37% [−92%, 25%], Figure 2) or high N (estimated change relative to low N supply: −0.36% [−8.5%, 6.3%], Figure 2) soil inoculation, but there were no statistical interactions between N supply and these soil treatments (Table 1). In contrast to plants grown with noninoculated growing medium, co‐inoculation did not affect BYDV‐PAV incidence when plants were grown with low N soil (estimated change relative to single inoculation: 24% [−75%, 164%]) or ambient N soil (estimated change relative to single inoculation: −27% [−80%, 32%]), leading to interactions between co‐inoculation and each of the soil treatments (Table 1, Figure 2). However, co‐inoculation reduced BYDV‐PAV incidence from 0.99 to 0.70 (−29% [−58%, −5.7%]) when plants were grown with high N soil (Figure 2). Soil treatments did not affect significantly CYDV‐RPV incidence (Figure 3, Table 2) or co‐infection incidence (Figure 4, Table 3).

### 3.3 Long‐term N‐enriched soils, N supply, and infection: effects on the host

**FIGURE 5:**
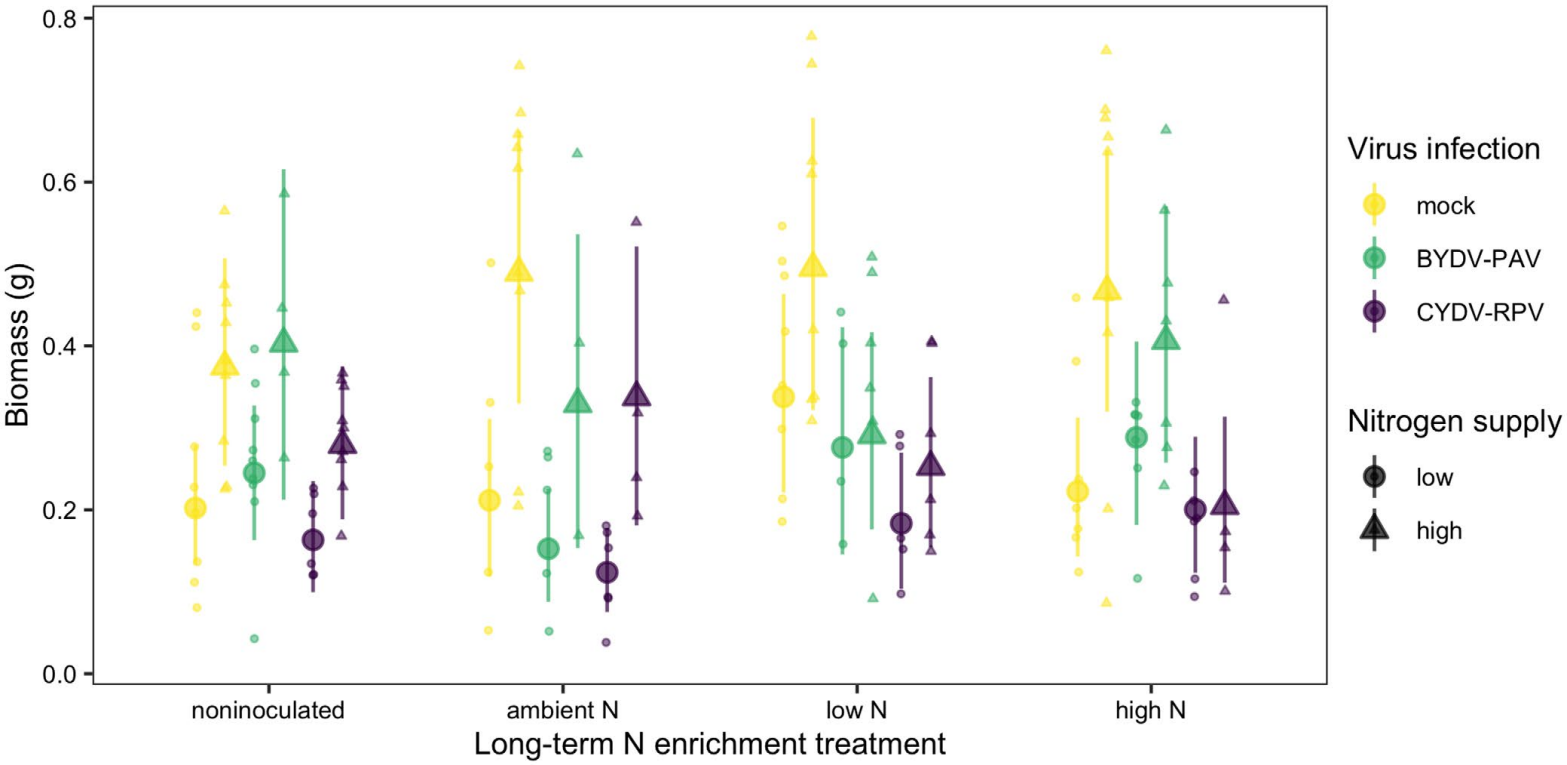
Aboveground biomass (g/plant) of plants grown with noninoculated growing medium, field soil exposed to long‐term ambient N, field soil exposed to long‐term low N, and field soil exposed to long‐term high N. Plants were grown with low or high N supply and were uninfected (mock inoculation) or infected with BYDV‐PAV, CYDV‐RPV, or both (co‐infection). Points and error bars are model‐estimated mean and 95% HDI. Small points are raw data

The average aboveground biomass of mock‐inoculated plants grown in noninoculated growing medium with low N supply was 0.20 g [0.13 g, 0.28 g] (Figure 5). High N supply increased biomass to 0.37 g, a 92% increase [4%, 192%] (Figure 5, Table 4). Infection and soil treatment did not significantly affect aboveground biomass (Table 4). CYDV‐RPV infection reduced aboveground biomass on average, but the 95% credible intervals included zero (Table 4). Only 10% of simulated datasets of the same sample sizes used in the experiment had significant effects of CYDV‐RPV on biomass. In contrast, sample sizes 10 times of those in the experiment produced significant effects in 72% of simulated datasets.

**TABLE 4:** Summary of linear regression of log‐transformed aboveground biomass (*n* = 154)

Variable^a^	Estimate	Std. error	95% CI		R‐hat
			Lower	Upper	
**Intercept** ^b^	**−1.62**	**0.19**	**−1.99**	**−1.25**	**1.00**
**N supply**	**0.62**	**0.26**	**0.12**	**1.13**	**1.00**
BYDV‐PAV infection	0.19	0.26	−0.31	0.70	1.00
CYDV‐RPV infection	−0.22	0.28	−0.78	0.34	1.00
Soil ambient N	0.03	0.30	−0.55	0.63	1.00
Soil low N	0.51	0.26	0.00	1.03	1.00
Soil high N	0.09	0.27	−0.44	0.63	1.00
N supply × BYDV‐PAV	−0.14	0.41	−0.93	0.66	1.00
N supply × CYDV‐RPV	−0.07	0.38	−0.81	0.67	1.00
Soil ambient N × N supply	0.23	0.39	−0.54	1.00	1.00
Soil low N × N supply	−0.24	0.37	−0.95	0.48	1.00
Soil high N × N supply	0.13	0.37	−0.59	0.84	1.00
Soil ambient N × BYDV‐PAV	−0.52	0.42	−1.35	0.30	1.00
Soil low N × BYDV‐PAV	−0.41	0.41	−1.22	0.39	1.00
Soil high N × BYDV‐PAV	0.06	0.38	−0.69	0.80	1.00
Soil ambient N × CYDV‐RPV	−0.31	0.43	−1.15	0.53	1.00
Soil low N × CYDV‐RPV	−0.40	0.41	−1.22	0.40	1.00
Soil high N × CYDV‐RPV	0.11	0.41	−0.69	0.91	1.00
Soil ambient N × N supply × BYDV‐PAV	0.04	0.63	−1.21	1.28	1.00
Soil low N × N supply × BYDV‐PAV	−0.18	0.59	−1.33	0.96	1.00
Soil high N × N supply × BYDV‐PAV	−0.26	0.56	−1.36	0.85	1.00
Soil ambient N × N supply × CYDV‐RPV	0.21	0.58	−0.93	1.36	1.00
Soil low N × N supply × CYDV‐RPV	0.02	0.56	−1.08	1.11	1.00
Soil high N × N supply × CYDV‐RPV	−0.66	0.57	−1.77	0.44	1.00

^a^ Variables with quantile‐based 95% credible intervals that omit zero are in bold.

^b^ Intercept factor levels: low N supply, mock inoculation, noninoculated growing medium.

**FIGURE 6:**
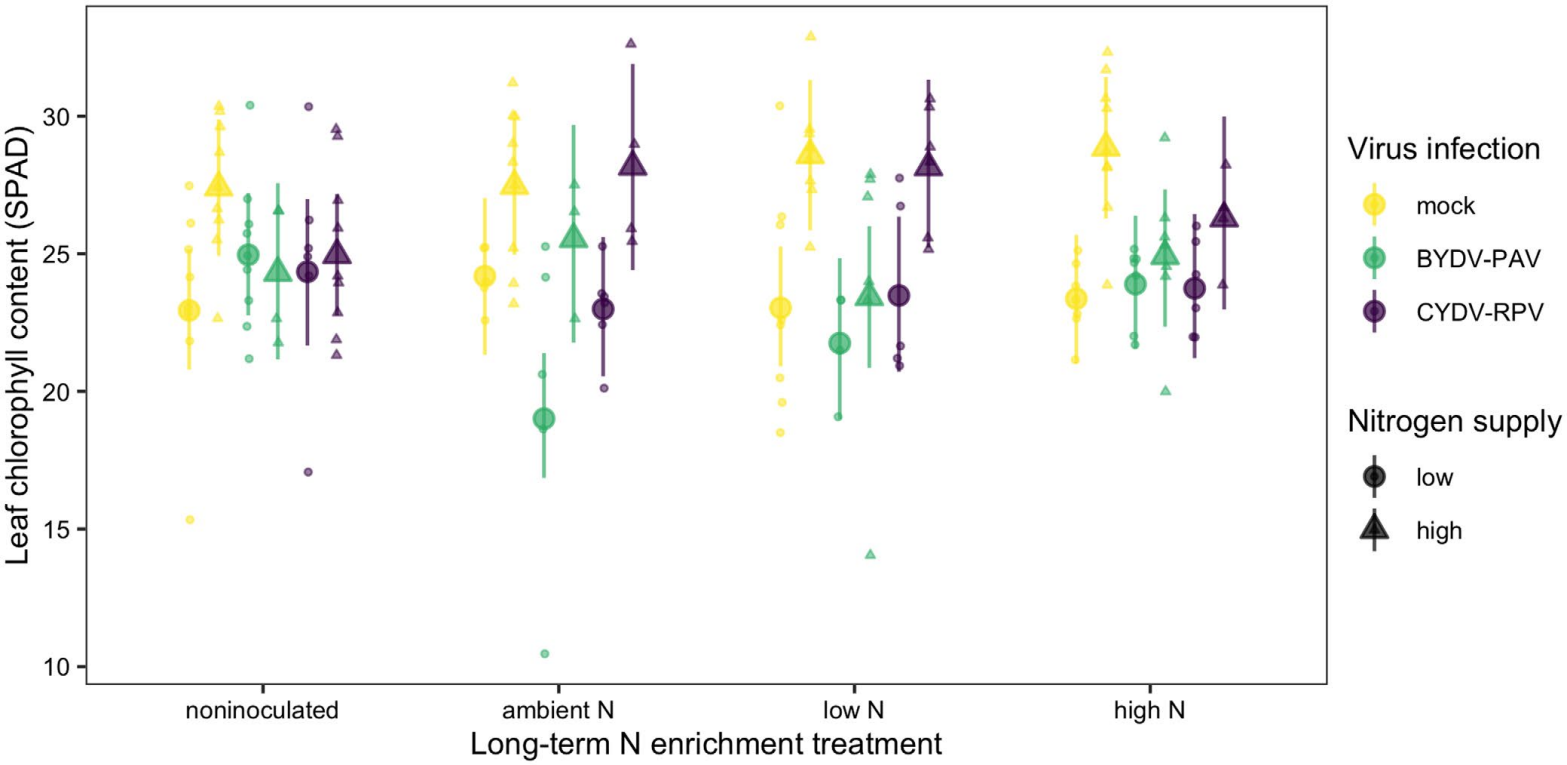
Leaf chlorophyll content (SPAD) of plants grown with noninoculated growing medium, field soil exposed to long‐term ambient N, field soil exposed to long‐term low N, and field soil exposed to long‐term high N. Plants were grown with low or high N supply and were uninfected (mock inoculation) or infected with BYDV‐PAV, CYDV‐RPV, or both (co‐infection). Points and error bars are model‐estimated mean and 95% HDI. Small points are raw data

The average leaf chlorophyll content of mock‐inoculated plants grown in noninoculated growing medium with low N supply was 23 SPAD [21 SPAD, 25 SPAD] (Figure 5). High N supply increased leaf chlorophyll content to 27 SPAD, a 20% increase [4%, 36%] (Figure 6, Table 5). Infection did not significantly affect leaf chlorophyll content of plants grown in noninoculated growing medium (Table 5), but BYDV‐PAV infection reduced leaf chlorophyll content from 24 SPAD to 19 SPAD, a 21% decrease [−34%, −8%], for plants grown with ambient N soil (Figure 6). However, when plants were grown with high N supply and ambient N soil, BYDV‐PAV infection did not affect leaf chlorophyll content (estimated change relative to no infection: −7% [−23%, 10%]), leading to a three‐way interaction among ambient N soil, N supply, and BYDV‐PAV infection (Table 5, Figure 6).

**TABLE 5:** Summary of linear regression of log‐transformed leaf chlorophyll content (*n* = 154)

Variable^a^	Estimate	Std. error	95% CI		R‐hat
			Lower	Upper	
**Intercept** ^b^	**3.13**	**0.05**	**3.03**	**3.23**	**1.00**
**N supply**	**0.18**	**0.07**	**0.05**	**0.32**	**1.00**
BYDV‐PAV infection	0.08	0.07	−0.05	0.22	1.00
CYDV‐RPV infection	0.06	0.07	−0.09	0.21	1.00
Soil ambient N	0.05	0.08	−0.10	0.21	1.00
Soil low N	0.00	0.07	−0.13	0.14	1.00
Soil high N	0.02	0.07	−0.12	0.16	1.00
N supply × BYDV‐PAV	−0.21	0.11	−0.42	0.01	1.00
N supply × CYDV‐RPV	−0.15	0.10	−0.35	0.04	1.00
Soil ambient N × N supply	−0.05	0.10	−0.25	0.15	1.00
Soil low N × N supply	0.04	0.10	−0.15	0.23	1.00
Soil high N × N supply	0.03	0.10	−0.16	0.22	1.00
**Soil ambient N × BYDV‐PAV**	**−0.33**	**0.11**	**−0.54**	**−0.11**	**1.00**
Soil low N × BYDV‐PAV	−0.14	0.11	−0.35	0.06	1.00
Soil high N × BYDV‐PAV	−0.06	0.10	−0.26	0.13	1.00
Soil ambient N × CYDV‐RPV	−0.11	0.11	−0.33	0.11	1.00
Soil low N × CYDV‐RPV	−0.04	0.11	−0.25	0.17	1.00
Soil high N × CYDV‐RPV	−0.04	0.11	−0.25	0.17	1.00
**Soil ambient N × N supply × BYDV‐PAV**	**0.37**	**0.17**	**0.05**	**0.69**	**1.00**
Soil low N × N supply × BYDV‐PAV	0.07	0.15	−0.24	0.36	1.00
Soil high N × N supply × BYDV‐PAV	0.04	0.15	−0.25	0.32	1.00
Soil ambient N × N supply × CYDV‐RPV	0.23	0.15	−0.07	0.53	1.00
Soil low N × N supply × CYDV‐RPV	0.12	0.15	−0.17	0.41	1.00
Soil high N × N supply × CYDV‐RPV	0.04	0.15	−0.25	0.34	1.00

^a^ Variables with quantile‐based 95% credible intervals that omit zero are in bold.

^b^ Intercept factor levels: low N supply, mock inoculation, noninoculated growing medium.

## 4 DISCUSSION

Human activities have dramatically increased N supply to terrestrial ecosystems, with consequences for plant communities, plant–pathogen interactions, and pathogen–pathogen interactions (Elser et al., 2007; Midolo et al., 2019; Smith, 2014; Veresoglou et al., 2013). Long‐term N enrichment can shift the composition and function of soil microbial communities (Huang et al., 2019; Klinger et al., 2016), which can mediate N availability, soil‐borne pathogens, and plant defenses (van der Heijden et al., 2008; Kuzyakov & Xu, 2013; Mauch‐Mani et al., 2017). However, the microbe‐mediated effects of long‐term N enrichment on aboveground pathogen communities and host–pathogen interactions have received little attention. Our experimental manipulation of long‐term N‐enriched soil inoculum, N supply, and virus infection suggests that long‐term N enrichment, potentially mediated by changes in the soil microbial community, can influence aboveground plant–pathogen and pathogen–pathogen interactions and mediate the impact of infection on plant traits. Specifically, ambient or low N soil mediated the effects of N supply and co‐inoculation on BYDV‐PAV infection incidence and BYDV‐PAV infection on plant chlorophyll content. Surprisingly, soil treatments did not affect aboveground plant biomass, and had different impacts on the incidence of two related viral pathogens.

Long‐term low N‐enriched soil reduced BYDV‐PAV incidence and counteracted the negative effects of N supply and co‐inoculation on BYDV‐PAV incidence. Few, if any, other studies have yet demonstrated that changes in soil microbes due to N enrichment may mediate the effects of N supply on a non‐soil‐borne plant pathogen. Of the three general mechanisms through which soil microbes can affect plant–pathogen interactions—modified access to resources (van der Heijden et al., 2008; Kuzyakov & Xu, 2013; Schimel & Bennett, 2004), promotion or suppression of pathogens in the soil (Berendsen et al., 2012; Lekberg et al., 2021; Schlatter et al., 2017), and induced plant defenses (van Loon et al., 1998; Mauch‐Mani et al., 2017; Pieterse et al., 2014)—the third is the most likely to apply to our study. Soil treatment did not significantly affect plant biomass and only mediated the effect of infection on leaf chlorophyll content, suggesting that potential shifts in soil microbes due to long‐term N enrichment did not generally affect plant access to resources. Measurements of plant and soil N content in future studies would help evaluate this conclusion more rigorously. Because BYDV‐PAV and CYDV‐RPV are obligately transmitted to plants by aphid vectors (D’Arcy & Burnett, 1995), soil microbes could only affect virus incidence indirectly via effects on the plant, ruling out the second mechanism. Induction of plant defenses could explain why BYDV‐PAV incidence was significantly lower when plants were grown with low N soil. Furthermore, ambient and low N soil negated the effects of co‐inoculation and N supply on BYDV‐PAV incidence and the combined effect of co‐inoculation and N supply on BYDV‐PAV incidence was subadditive. These interactions are consistent with these factors affecting BYDV‐PAV incidence through a common mechanism, such as host defenses. Soil treatments did not affect CYDV‐RPV incidence. BYDV‐PAV had a higher prevalence at the field site in a single survey (see Section 2), which may indicate more frequent indirect interactions between BYDV‐PAV and soil microbes at the site. However, long‐term monitoring would be needed to assess whether BYDV‐PAV consistently occurs at a higher prevalence than CYDV‐RPV.

Increased N supply reduced BYDV‐PAV incidence in plants grown in noninoculated growing medium. Higher N availability may have decreased plant susceptibility to infection, which has been demonstrated for other, usually necrotrophic, pathogens (Dordas, 2009; Vega et al., 2015). In a field experiment, N supply decreased BYDV‐PAV incidence only when P supply was high, which suggests that the stoichiometry of nutrient supply (e.g., N:P) influences plant–pathogen interactions rather than the absolute supply (Borer et al., 2014). Our results may, therefore, suggest that BYDV‐PAV infection was more successful when plants were grown with higher P:N supply, perhaps due to higher within‐host virus replication, as has been demonstrated in aquatic systems with P:C stoichiometry (Clasen & Elser, 2007; Frost et al., 2008). However, studies that have measured within‐host BYDV‐PAV titer have not found a positive effect of P or P:N (Kendig et al., 2020; Lacroix et al., 2017; Rúa et al., 2013; Whitaker et al., 2015). The mechanism behind reduced BYDV‐PAV incidence with higher N supply, therefore, requires a closer examination of plant defenses, within‐host dynamics, and plant–vector interactions. Nitrogen supply no longer reduced infection incidence when plants were co‐inoculated. Interestingly, N supply and co‐inoculation also interacted to affect virus incidence in a study conducted by Lacroix et al. (2014), except that the interaction affected CYDV‐RPV incidence rather than BYDV‐PAV. These results suggest that BYDV‐PAV and CYDV‐RPV may interact within hosts and that nutrient supply may modify their interactions, analogous to pathogens in animal and human systems (Smith, 2014; Smith & Holt, 1996).

The long‐term N enrichment treatments, which have been previously shown to shape the soil microbial community (Fierer et al., 2012; Schlatter et al., 2013), modified plant–pathogen interactions. Only soil exposed to multiple decades of low N enrichment significantly reduced BYDV‐PAV incidence. Ambient N soil caused a weaker (and nonsignificant) reduction in BYDV‐PAV incidence while all plants grown with high N soil became infected with BYDV‐PAV (unless they were co‐inoculated). If soil microbes affected BYDV‐PAV incidence through induced plant defenses, these results suggest that long‐term N enrichment could indirectly influence plant defenses via the soil microbial community. Specifically, long‐term low N‐enriched soil may induce plant defenses while long‐term high N‐enriched soil may not. This result contrasts with previous studies that have demonstrated that higher nutrient availability increased the disease suppressive activity of plant‐associated microbes (Berg & Koskella, 2018; Wiggins & Kinkel, 2005), but a nonmonotonic relationship between N enrichment and microbe‐induced plant defenses is possible. We also found that BYDV‐PAV infection only reduced leaf chlorophyll content when plants were grown with ambient N soil. This result suggests that not only may the microbial community mediate the success of BYDV‐PAV infection but it may also mediate some of the symptoms of infection experienced by infected plants. We do not know whether long‐term N enrichment shifted the composition, function, or both of the soil microbial communities (Chen et al., 2019; Klinger et al., 2016; Leff et al., 2015), but subsequent studies could characterize microbial taxa and function associated with changes in BYDV‐PAV infection.

We selected the host species and virus species because of their importance for agriculture (Mckirdy et al., 2002; Riedell et al., 2007) and the wide knowledge base provided by previous studies (Baltenberger et al., 1987; Carrigan et al., 1983; Erion & Riedell, 2012; Lacroix et al., 2014; Power et al., 1991). However, the host species does not naturally co‐occur with the soil microbial communities sampled in this study, which may have limited the observed effects of soil treatment on plant growth and plant–pathogen interactions (Essarioui et al., 2020). Indeed, studies that have used co‐occurring plant species and soil microbial communities have found statistically significant effects of the N enrichment history of field soil on plant biomass (Johnson, 1993; Weese et al., 2015). Therefore, our study may have isolated the effects of soil microbes that are generalists or that affect plant–pathogen interactions without requiring co‐evolved interactions. A follow‐up study may consider exploring the relationship of nutrients, plant pathogens, and soil microbes from communities in which the species naturally occur. In addition to no effect of soil treatment on plant biomass, we also found no effect of infection on plant biomass. BYDV‐PAV and CYDV‐RPV infections typically reduce plant biomass (Baltenberger et al., 1987; Erion & Riedell, 2012), but the effects of plant pathogens depend on environmental conditions (Barrett et al., 2009). BYDV‐PAV and CYDV‐RPV infections may have affected the plants in ways that we did not measure, such as root growth or mineral nutrient concentrations (Riedell et al., 2007). Additionally, based on simulated datasets, our study’s sample sizes may have impeded our ability to detect small changes in biomass. Although we did not measure plant or soil N content, high N supply increased the biomass and chlorophyll content of mock‐inoculated plants grown in noninoculated growing medium (i.e., control plants), suggesting that manipulation of N supply was successful.

B/CYDVs are vectored by aphids, which may mediate the effects of soil nutrients and soil microbes on plant–pathogen and pathogen–pathogen interactions. For example, increasing N supply to plants can increase or decrease the length of time that aphids feed (Bogaert et al., 2017; Nowak & Komor, 2010), which affects the probability that viruses successfully infect plants (Power et al., 1991). Soil microbial communities also can influence the feeding behavior and population dynamics of aphids (Pineda et al., 2010). For example, soil microbes can increase or decrease the weight, body size, and intrinsic growth rate of aphids (Hackett et al., 2013; Hol et al., 2010; Pineda et al., 2012). Higher aphid population densities may increase the co‐infection incidence of B/CYDVs (Seabloom et al., 2009). In addition, N supply and soil microbial communities may interact to affect aphid vectors because the effects of microbes on insect herbivores tend to be stronger when plants experience abiotic stress (Pineda et al., 2013). For instance, a previous study demonstrated that other soil‐dwelling organisms, nematodes, affected aphid population growth rates and plant preference only when N was limited (Kutyniok et al., 2014). To evaluate the role of aphids in pathogen responses to N supply and soil microbial communities, future studies could investigate the effects of N and microbes on aphid feeding duration in the lab and aphid population dynamics in the field.

Our experiment provided a novel demonstration that long‐term N‐enriched soil may affect the incidence of an insect‐vectored virus, BYDV‐PAV, in a plant host, *A*. *sativa*, through shifts in the soil microbial community. Because soil treatments mediated the effects of contemporary N supply, and the N enrichment history of soil impacted both incidence and chlorophyll of infected plants, our results suggest that inferences about how N enrichment modifies plant pathogens based on laboratory experiments will depend on the role of soil microbes. Furthermore, our results suggest that high N enrichment could reduce the pathogen‐suppressive effects of soil microbes for some plant–pathogen pairs, potentially leading to more widespread infection under field conditions with elevated N supply. This work demonstrates the important indirect role of soil microbial communities for infection outcomes, pointing to an exciting new frontier: examining the generality and context dependence of these results, and the indirect role of soil microbes in the high variation in plant–microbe (Smith & Goodman, 1999) and plant–pathogen (Hoffland et al., 2000) interactions.

## CONFLICT OF INTEREST

The authors declare no conflict of interest.

## AUTHOR CONTRIBUTIONS

**Casey A. Easterday:** Conceptualization (equal); Formal analysis (equal); Investigation (equal); Methodology (equal); Writing – original draft (equal); Writing – review & editing (equal). **Amy Kendig:** Conceptualization (equal); Formal analysis (equal); Methodology (equal); Writing – original draft (equal); Writing – review & editing (equal). **Christelle Lacroix:** Conceptualization (equal); Methodology (equal); Supervision (equal); Writing – review & editing (equal). **Eric Seabloom:** Conceptualization (equal); Funding acquisition (equal); Methodology (equal); Supervision (equal); Writing – review & editing (equal). **Elizabeth Borer:** Conceptualization (equal); Funding acquisition (equal); Methodology (equal); Supervision (equal); Writing – review & editing (equal).

### OPEN RESEARCH BADGES

This article has earned an Open Data Badge for making publicly available the digitally‐shareable data necessary to reproduce the reported results.

## Data Availability

Data and code are available through the Environmental Data Initiative Data Portal: https://doi.org/10.6073/pasta/5620600a9d7d7bec7855ba1999a37ded.

## APPENDIX 1

### Additional methods

**TABLE A1:** Modified Hoagland solution used to manipulate N supply rate. The low and high concentrations of NH_4_NO_3_ are 0.2% and 10% of half‐strength Hoagland solution, respectively

Compound	Molar mass	Concentration (μM)
K_2_SO_4_	174.25	1250
MgSO_4_·7H_2_O	246.48	1000
KH_2_PO_4_	136.09	1
CaSO_4_·2H_2_O	172.17	2000
NH_4_NO_3_	80.04	7.5 (375 for high N)
KCl	74.56	25
H_3_BO_3_	61.83	12.5
MnSO_4_·H_2_O	169.02	1
ZnSO_4_·7H_2_O	278.56	1
CuSO_4_·5H_2_O	249.69	0.25
H_2_MoO_4_·(H_2_O)	161.95	0.25
NaFeEDDHA (6% Fe)	434.8	10

**TABLE A2:** Plants with virus infections inconsistent with their inoculation treatment. Total sample sizes are in parentheses

Inoculation treatment	BYDV‐PAV	CYDV‐RPV
Mock (77)	2	12
CYDV‐RPV (78)	3	
BYDV‐PAV (74)		14
